# Combined use of apatinib mesylate and vinorelbine versus vinorelbine alone in recurrent or metastatic triple-negative breast cancer: study protocol for a randomized controlled clinical trial

**DOI:** 10.1186/s13063-020-04342-x

**Published:** 2020-05-24

**Authors:** Shuo Wu, Liang Zhang, Huan Li, Junnan Xu, Cui Jiang, Tao Sun

**Affiliations:** 1 Department of Medical Oncology, Cancer Hospital of China Medical University, Liaoning Cancer Hospital & Institute, Shenyang, 110042 Liaoning Province China; 2 Key Laboratory of Liaoning Breast Cancer Research, Cancer Hospital of China Medical University, Liaoning Cancer Hospital & Institute, Shenyang, 110042 Liaoning Province China

**Keywords:** Triple-negative breast cancer, Apatinib, Vinorelbine, Advanced breast cancer, Metastatic

## Abstract

**Background:**

The emergence of new molecular targeted drugs provides new prospects for the treatment of advanced breast cancer; the future therapeutic trend includes chemotherapy combined with molecular targeted therapy. Apatinib mesylate, a novel, small anti-angiogenic agent, highly selectively inhibits the activity of vascular endothelial growth factor receptor-2 tyrosine kinase. Apatinib mesylate also blocks the signaling of vascular endothelial growth factor binding to its receptor, thereby strongly inhibiting tumor angiogenesis and exerting an anti-tumor effect. However, there have been no reports of a randomized controlled clinical trial of apatinib combined with vinorelbine for the treatment of triple-negative breast cancer (TNBC). We will compare the therapeutic effect of vinorelbine alone or in combination with apatinib mesylate, in patients with recurrent or metastatic TNBC in North China who have received at least two drug treatments, including anthracyclines and taxanes.

**Methods/analysis:**

This study is a triple-blind, randomized, placebo-controlled, parallel-group clinical trial. We plan to include 238 female patients with locally recurrent or metastatic TNBC, admitted to the Liaoning Cancer Hospital & Institute, Northeast China. All enrolled patients will be randomized to oral vinorelbine alone (40 mg, thrice a week (Mondays, Wednesdays, and Fridays) in each 3-week cycle), or in combination with oral apatinib mesylate (500 mg, once daily in each 3-week cycle). Radiographic assessment will be performed every 6 weeks for 36 weeks and every 9 weeks thereafter. The primary outcome is progression-free survival and secondary outcomes include overall survival, disease control rate, objective response rate, and incidence of adverse events at grades 3 and 4, as defined by the National Cancer Institute Common Toxicity Criteria Version 4.0. Outcome measures will be evaluated at baseline (< 2 weeks before starting treatment), every 6 weeks during treatment, and at 4 weeks and every 3 months after treatment discontinuation.

**Discussion:**

Based on the data from this trial, we hope to identify a treatment plan that is suitable for female patients with TNBC, who have been treated with anthracyclines and taxanes, in Northeast China.

**Trial registration:**

ClinicalTrials.gov: NCT03932526. Registered on 30 April 2019.

## Introduction

### Background

The International Agency for Research on Cancer predicts that the number of cancers in the future will increase at an annual rate of 3–5%. The incidence of cancer in low-income and middle-income countries is extremely higher than that in developed countries [1]. Breast cancer is one of the most highly prevalent malignant tumors, with approximately 13 million people newly diagnosed each year. With a mortality rate of 20–30%, breast cancer is a common cause of death among women, accounting for 400,000 deaths annually. In developing countries, breast cancer has become the leading cause of death in women [2].

Triple-negative breast cancer (TNBC) is a subtype of breast cancer with an absence of estrogen receptors (ER), progesterone receptors (PR), and human epidermal growth factor receptor 2 (HER-2). It accounts for 10–17% of all breast cancers [3], and is characterized by high heterogeneity, high invasiveness, low survival rate, early recurrence, and metastasis. There is a lack of effective treatment for TNBC, and the prognosis is poor [4, 5].

Despite significant advances in breast cancer treatment, approximately 15% of patients are diagnosed at an advanced stage of breast cancer. According to staging, grading, and choice of treatment, 20–80% of all patients with invasive breast cancer will eventually relapse and require subsequent treatment [6]. Chemotherapy is the main treatment for early and advanced breast cancer, and the most effective drugs include anthracyclines and taxanes [7]. However, the increasing use of anthracyclines and taxanes in the early stage of the disease makes the choice of a second-line therapy difficult, and drug resistance often limits the choice of treatment regimens [8]. Endocrine therapy, anti-HER-2 targeted therapy, and chemotherapy cannot achieve satisfactory outcomes in TNBC, as there is no corresponding hormone receptor or HER-2 expression.

Some progress has been made in the field of TNBC therapy with the use of immunological checkpoint inhibitors. PD-1 or PD-L1 monoclonal antibody, the most common immunological checkpoint inhibitor, which targets either PD-1 or PD-L1, can block this binding and boost the immune response against cancer cells. Currently, only PD-L1 monoclonal antibody - atezolizumab (Tecentriq®) and PD-1 monoclonal antibody - pembrolizumab (Keytruda®) have been approved for use by the US Food and Drug Administration (FDA). The combination of pembrolizumab plus chemotherapy as a neoadjuvant therapy for early TNBC can significantly improve pathologic complete remission [9]. Relative to chemotherapy, pembrolizumab is used as a second-line or third-line treatment for patients with metastatic TNBC, but it does not significantly improve overall survival (OS) [10].

Results from the IMpassion130 trial (a clinical, phase III, double-blind randomized trial reported in 2016 [11], with data updated in 2019 [12]) indicated that the combination of atezolizumab and albumin paclitaxel as a first-line treatment for advanced TNBC yielded better progression-free survival than the use of paclitaxel plus placebo.

Unfortunately, no approval has been given for clinical trials on checkpoint inhibitors for TNBC in China; furthermore, no such drug is available in clinical practice. Therefore, this study was designed to determine the effect of the combination of apatinib (a small-molecule, anti-angiogenic targeted drug) and vinorelbine (a semi-synthetic, vinblastine alkaloid antineoplastic drug), two types of drugs that have been used clinically in China, in the treatment of TNBC.

Combination chemotherapy with vascular endothelial growth factor (VEGF) and VEGF receptor (VEGFR) target inhibitors is one of the most promising regimens for advanced breast cancer [13]. Apatinib mesylate (Aitan) is a novel, small anti-angiogenic agent that highly selectively inhibits the activity of VEGFR-2 tyrosine kinase, and blocks the signaling of VEGF binding to its receptor. Thus, it strongly inhibits tumor angiogenesis and exerts anti-tumor effects. While the clinical use of apatinib in breast cancer has been reported, the relevant research has focused mainly on its safety and efficacy in breast cancer expressing different receptors [13, 14]. The administration of apatinib in patients with TNBC, at an initial dose of 750 mg/day, resulted in mean progression-free survival (mPFS) and mean OS (mOS) of 4.6 and 8.3 months, respectively. A smaller dose (500 mg/day) resulted in mPFS and mOS of 3.3 and 10.6 months, respectively [14]. In cases of advanced breast cancer, oral apatinib was used after first-line or second-line treatment failure. The objective response rate (ORR) was 40.0%, the disease control rate (DCR) was 75.0%, and the median time to progression (mTTP) was 12 months [13].

Vinorelbine is mainly used for the treatment of non-small cell lung cancer and metastatic breast cancer. A previous multicenter clinical trial [15] used vinorelbine combined with cisplatin, capecitabine, or tegafur for the treatment of recurrent and metastatic breast cancer that occurred after treatment with anthracyclines and taxanes. The effective rate was 61.0%, with a complete response (CR) of 4.9%. This combination therapy was highly effective for multiple and single metastatic lesions, and moreover, the therapeutic efficacy was better in multiple metastatic lesions. The short-term effect was fair, with tolerance to toxicity and good safety. Studies have reported the use of vinorelbine combined with 5-fluorouracil among patients with advanced metastatic breast cancer (following treatment failure with anthracycline/taxane), and an effective rate of 17.4–46.0% [16–18]. In patients with metastatic TNBC, vinorelbine or gemcitabine combined with cisplatin is preferred after anthracycline/taxane treatment failure. The objective response rates of the experimental group and the control group were 45.45% and 46.15%, respectively, and the DCRs in the two groups were 77.27% and 80.77%, respectively. In the experimental group, the time to progression (TTP) was 2.0–18.0 months, and the mTTP was 5.0 months (95% confidence interval (CI), 3.28–6.72). In the control group, the TTP was 1.8–18.5 months, with an mTTP of 5.2 months (95% CI, 3.33–7.07) [19].

### Study features and objectives

While previous cohort studies [13, 20, 21], retrospective observations [22–25], and case reports [26, 27] have addressed the clinical treatment of TNBC with apatinib or vinorelbine (Table 1), there have been no reports of a randomized, controlled clinical trial in North China. As *BRCA*-positive patients would have poor responses to the therapeutic methods designed in this study, *BRCA*-negative female patients with locally recurrent or metastatic TNBC, who have previously received anthracycline and taxane treatment, will be the target population in this trial. We will compare the therapeutic effect of vinorelbine used alone or in combination with apatinib mesylate, for the treatment of recurrent or metastatic TNBC, with the aim of providing clinical evidence for multi-line treatment options.

**Table 1:** Recent clinical studies conducted within the past 5 years on treatment with apatinib or vinorelbine in patients with TNBC pretreated with chemotherapy or radiotherapy

Study	Design	Subjects	Assignment	Treatment	Outcome measures	Conclusion
Treatment with apatinib						
Hu et al. [13]	Phase II cohort study	84 patients previously treated with anthracycline and/or taxane: 25 in phase IIa trial: 59 in phase IIb trial	–	Phase IIa trial: apatinib, daily dose of 750 mg Phase IIb trial: apatinib, daily dose of 500 mg	ORR and CBR were 10.7% and 25.0%, respectively. Median PFS and OS were 3.3 and 10.6 months, respectively	An apatinib dose of 500 mg rather than 750 mg is the recommended starting dose for heavily pretreated patients with mTNBC with a measurable rate of partial response and PFS
Li et al. [22]	Retrospective analysis	44 patients with advanced TNBC with failed first-line or second-line therapy	Apatinib + capecitabine or capecitabine alone	Apatinib (500 mg) was orally administered daily on days 1–28 of each 4-week cycle and/or capecitabine (12,500 mg/m^2^) was orally taken twice daily for 14 days followed by a 7-day rest period until disease progression	PFS, ORR (CR + PR), DCR (CR + PR + SD), and toxicity For apatinib + capecitabine or capecitabine alone: PFS, 5.5, 3.5 months; ORR, 40.9%, 13.4%; DCR, 68.2%, 31.8%	Treatment with a combination of apatinib and capecitabine can achieve a better efficacy and similar rate of serious adverse events compared with capecitabine alone, as the third-line treatment for advanced TNBC
Hu et al. [26]	Case report	A female patient with stage IV TNBC. She had previously undergone whole-brain radiation therapy. Paclitaxel, platinum, UTD1, capecitabine, gemcitabine, vinorelbine, and single-agent apatinib were then administered as first-line to fifth-line therapies	–	Treatment with apatinib + CPT-11 + S-1 as the sixth-line therapy	Alleviation of the brain edema was achieved, and this was maintained for 7 months	Apatinib in combination with CPT-11 + S-1 to treat refractory BMs in a patient with TNBC
Zhou et al. [27]	Case report	A female patient with triple-negative spindle cell carcinoma	–	After treatment failure with bevacizumab combined with albumin-bound paclitaxel and cisplatin, the patient was treated with apatinib	Apatinib was more effective than bevacizumab for this patient	Apatinib is a safe and effective drug for treating advanced spindle cell breast carcinoma, especially in patients who have a prior experience of chemotherapy failure, or a poor physical condition
Treatment with vinorelbine						
Rodler et al. [20]	Phase I cohort study	50 patients with advanced TNBC	–	A 3 + 3 dose escalation design evaluated veliparib and vinorelbine, followed by veliparib monotherapy	Median PFS in 50 patients was 5.5 months	Veliparib (300 mg, twice daily) combined with cisplatin and vinorelbine is well-tolerated with encouraging response rates
Zhang et al. [21]	Prospective cohort study	44 patients with metastatic TNBC pretreated with anthracyclines and/or taxanes	–	Biweekly combination of vinorelbine and oxaliplatin (NVBOX)	ORR 31.6%, median PFS 4.3 months, OS 12.6 months	A biweekly NVBOX regimen is effective and well-tolerated as a second-line or third-line treatment for patients with mTNBC
Wang et al. [23]	Retrospective analysis	48 female patients with TNBC	–	Patients were given vinorelbine plus cisplatin (NP group, *n* = 22) or gemcitabine plus cisplatin (GP group, *n* = 26)	The ORR, DCR, and median TTP were 45.5%, 77.3%, and 5 months, respectively, in the NP group, and 46.2%, 80.8%, and 5.2 months, respectively, in the GP group A lower incidence of thrombocytopenia and rash, and a higher incidence of phlebitis was found in the NP group compared to the GP group	Both the NP and GP regimens are active and tolerated in cases of metastatic TNBC with anthracycline and/or taxane resistance. These regimens may be used as a salvage treatment for metastatic TNBC
Du et al. [24]	Retrospective analysis	48 patients with metastatic TNBC pretreated with anthracyclines and one taxane	–	22 patients were treated with vinorelbine plus platinum (NP), and 26 patients with vinorelbine plus capecitabine (NX)	Total: ORR, 20.8%; CBR, 43.8%; median PFS, 4.4 months; median OS, 15.5 months ORR and PFS were better in the NP arm versus NX	Vinorelbine-based combination chemotherapy shows moderate efficacy in the treatment of metastatic TNBC, and has manageable toxicity. The NP regimen shows potential superiority over the NX regimen.
Li et al. [25]	Retrospective analysis	41 patients with advanced TNBC (pretreated with anthracyclines and/or taxanes, before or after surgery) in whom disease progressed after a certain period of time	–	Treatment with vinorelbine plus platinum (NP) regimen	ORR, 34.1%; CR, 7.3%; partial response, 26.8%; stable disease, 34.1%; median OS, 18.9 months; PFS, 6.7 months	The NP regimen showed clinical activity in patients with metastatic TNBC, and the toxicity was acceptable and manageable

*PFS* median progression-free survival, *ORR* objective response rate, *DCR* disease control rate, *BM* brain metastasis, *TNBC* triple-negative breast cancer, *CBR* clinical benefit rate, *OS* overall survival, *CR* complete response, *PR* partial response, *SD* stable disease, *TTP* time to progression

## Methods and analysis

### Study design

This study is a triple-blind, randomized, placebo-controlled, parallel-group clinical trial. A sample of 238 female patients with recurrent or metastatic TNBC, who have been pretreated with at least one chemotherapy regimen (including anthracyclines and taxanes), will be recruited. The baseline characteristics, therapeutic schedules, and outcomes of enrolled patients will be documented and reported in accordance with the Consolidated Standards of Reporting Trials (CONSORT) statement [28]. Patient data in each center will be collected by an electronic data capture system.

**Fig. 1:**
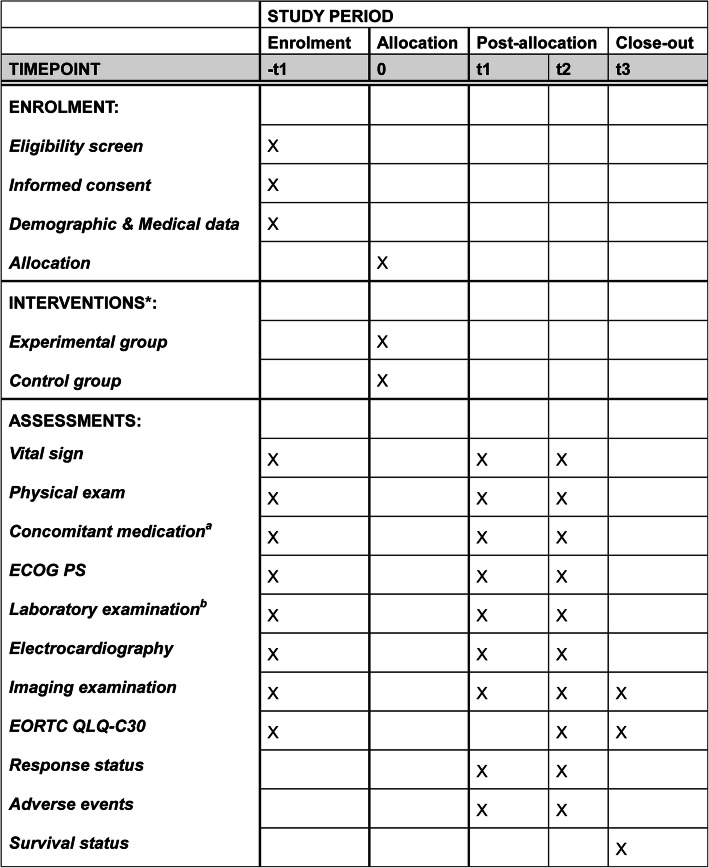
Standard Protocol Items: Recommendations for Interventional Trials (SPIRIT). Timepoint (t): -t1: baseline evaluations (conducted within 2 weeks of the start of protocol therapy); t0: random allocation; t1: during treatment (evaluations will be conducted every 6 weeks (two cycles)); t2: patients will be monitored for new or existing AEs at 4 weeks after treatment discontinuation; t3: follow up for survival will be monitored every 3 months after treatment discontinuation until patient death or study completion. *Eligible patients will be randomly assigned to receive either oral vinorelbine plus placebo (control group) or oral vinorelbine combined with apatinib mesylate (experimental group). ^a^ Concomitant medication includes opioid analgesics and new anticancer treatment. ^b^ Laboratory examinations include hematology (hemoglobin, white blood cell count, neutrophil count, and platelet count); blood biochemical tests (total bilirubin, alanine aminotransferase, aspartate aminotransferase, alkaline phosphatase, serum creatinine, total protein, Na^+^, K^+^, Mg^2+^, Cl^−^, Ca^2+^, urea, and pregnancy test (if applicable); and tumor marker detection (breast cancer-associated antigen CA153 and carcinoembryonic antigen). ECOG PS, Eastern Cooperative Oncology Group performance status; EORTC QLQ-C30, the European Organization for Research and Treatment of Cancer Quality of Life Questionnaire Core-30

**Fig. 2:**
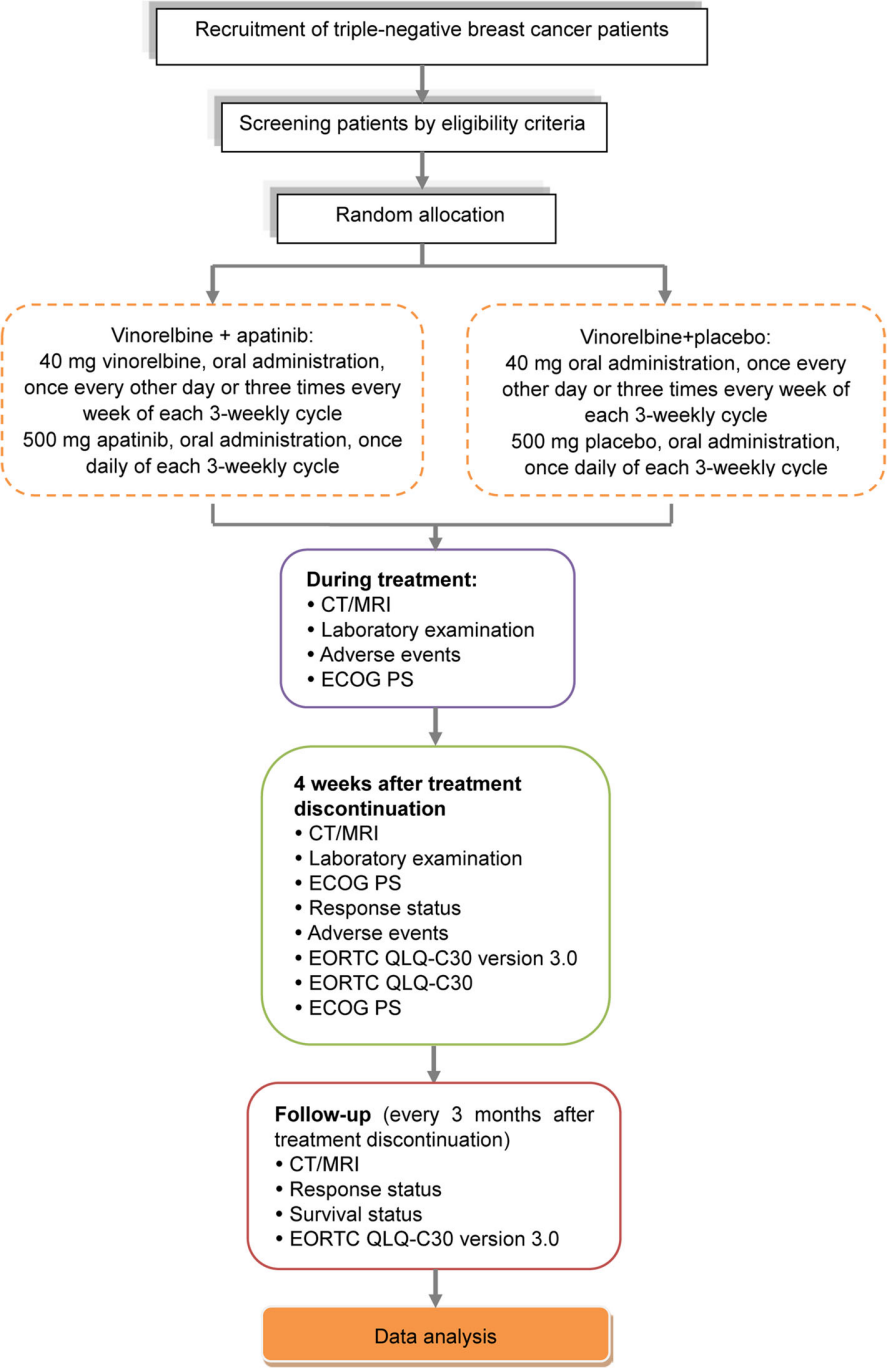
Schedule of enrollment, interventions, and assessments. ECOG PS, Eastern Cooperative Oncology Group performance status; EORTC QLQ-C30, version 3, the European Organization for Research and Treatment of Cancer Quality of Life Questionnaire Core-30; CT, computed tomography; MRI, magnetic resonance imaging

All enrolled patients will be randomly assigned to receive either oral apatinib mesylate in combination with vinorelbine or oral vinorelbine plus placebo, until disease progression or other criteria indicate the need for the termination of drug administration. A schedule of enrollment, interventions, and assessments is shown in Fig. 1 and a trial flowchart is shown in Fig. 2. The study protocol follows the Standard Protocol Items: Recommendations for Interventional Trials (SPIRIT) guidance for protocol reporting (Additional file 1) [29].

### Study participants

The study participants will be recruited from the Liaoning Cancer Hospital & Institute in Northeast China.

#### Inclusion criteria

The inclusion criteria are:

1. Female patients with recurrent or metastatic TNBC, confirmed by histological or cytological examination.
2. Age ≥ 18 years.
3. At least one extracranial measurable site of disease according to Response Evaluation Criteria In Solid Tumors (RECIST) version 1.1 criteria [30] and an Eastern Cooperative Oncology Group performance status (ECOG-PS) score of 0–2.
4. Expected survival ≥ 12 weeks.
5. Negative for ER/PR: where ER < 1% indicates positive and PR < 1% indicates positive. Negative for HER-2 refers to: immunohistochemical analysis (IHC)1+ indicates HER-2 negative, IHC2+ indicates an uncertain case of HER-2, according to specific diagnostic criteria (the *Breast cancer HER-2 detection guide, 2014 edition*) [31]. Further, the in-situ hybridization (ISH) method will be used to detect HER-2 gene amplification for further diagnosis.
6. All patients will be tested for bone marrow capacity, liver, and renal function within 7 days prior to enrollment, and will meet the following criteria:
  - Routine blood test: absolute neutrophil (ANC) count ≥ 1.5 × 10^9^/L; hemoglobin ≥ 9.0 g/dL; platelet count ≥ 80 × 10^9^/L;
  - Liver function: total bilirubin ≤ 1.5 times the upper limit of normal (ULN); alanine aminotransferase (ALT) and aspartate aminotransferase (AST) ≤ 2.5 × ULN (patients with liver metastasis ≤ 5 × ULN); alkaline phosphatase ≤ 4 × ULN;
  - Renal function: serum creatinine ≤ 1.5 × ULN.
7. Previous use of anthracyclines and/or taxanes.
8. The medication history of vinorelbine meets one of the following conditions:
  - No history of, or irregular vinorelbine use (the standard use of vinorelbine is defined based on the appropriate use of the medication as prescribed, for at least two cycles);
  - Patients with advanced breast cancer, who receive a standardized regimen of vinorelbine for 6 months and have no cancer progression; they also have not used vinorelbine within 6 months prior to the first administration.
9. Female patients of childbearing age must take adequate contraceptio, otherwise they must be proven to be infertile, that is:
  - Patients over the age of 50 years are confirmed to have menstruation-deficient menopause for at least 12 months after stopping all exogenous hormone treatments;
  - Under the age of 50 years; on this basis, it is also necessary to prove that the levels of progesterone and follicle stimulating hormone are in the postmenopausal range of the research institution;
  - Women undergoing irreversible sterilization operations (hysterectomy, bilateral oophorectomy, bilateral salpingectomy, etc.) are negative for pregnancy and are not lactating before administration.
10. No history of serious heart, lung, liver, or kidney diseases.
11. Provision of written informed consent.

#### Exclusion criteria

The exclusion criteri are:

1. The patients are undergoing current administration of anticancer therapies, or are attending other clinical trials.
2. History or current evidence of brain metastasis, including leptomeningeal involvement.
3. Patients with a BRCA mutation who have poor response to the therapeutic regimen.
4. Patients with severe vascular diseases, including unstable angina, myocardial infarction, or severe arrhythmia in the past 6 months.
5. History of HIV infection or active chronic hepatitis B or C.
6. Patients with other serious infectious diseases.
7. Patients positive for ER/PR/HER-2.
8. Patients with allogeneic organ transplants requiring immunosuppressive therapy.
9. History of other malignant tumors within 5 years, except for cured cervical carcinoma in situ or basal cell carcinoma of the skin.
10. Other destabilizing factors (such as drug abuse and medical, psychological, or social conditions) that may interfere with patient compliance or have an impact on the trial results.
11. Allergy to target drugs or allergy to related drugs applied in the trial.
12. Pregnant or lactating women.

### Recruitment

Patients will be recruited based on their diagnosis at the Department of Breast Medicine, Liaoning Provincial Cancer Hospital in China, which has a sufficient number of patients with advanced breast cancer. Patients will be screened for eligibility by research staff to ensure all inclusion and exclusion criteria are met. The research staff identifying patients for inclusion into the study will be blinded to the study objectives.

### Randomization

A professional, independent statistician, who will not be involved in the recruitment process of the study, will randomize the participants. The statistician will use the Statistical Analysis System (SAS 9.1) software to generate a randomization sequence list, and assign each patient a serial number. Treatment allocations will be sealed in opaque envelopes prepared by research assistants who will not be involved in the recruitment process. These envelopes will be secured in a double-locked cabinet by another investigator who will not be involved in the trial.

### Blinding

The patients, investigators, and the statistician will be unaware of the treatment allocation until the end of the trial. The Office of Data Quality will provide the randomization assignment list to the Jiangsu Hengrui Pharmaceutical Co., Ltd., China, which will provide the blinded capsules. Nurses who will not participate in the trial will dispense drugs to eligible patients based on their allocation sequence.

In the case of an emergency, the investigators can promptly determine the medication that the patients are receiving, to ensure timely and correct medical treatment. If unblinding is necessary, study staff will contact the Office of Data Quality.

### Drug administration

Vinorelbine plus apatinib group: a 40 mg vinorelbine tartrate soft capsule (brand name Navelbine, registration number H20140657; Pierre Fabre Medicament, Boulogne, France) is administered orally in the morning (at least 1 h before, or at least 1 h after meals), three times a week (Mondays, Wednesdays, and Fridays), for a continuous 21-day cycle. A 500 mg apatinib mesylate tablet (brand name Aitan; state medical permission number. H20140103), is administered orally once a day for a continuous 21-day cycle.

Vinorelbine plus placebo group: oral administration of vinorelbine will be the same as in the vinorelbine plus apatinib group. In addition, the patients will be given oral placebo (starch as an ingredient). The placebo appearance, including shape, size, color and weight, taste, labeling, and packing is the same as that of the apatinib mesylate tablets.

The placebo and the apatinib will be manufactured by Jiangsu Hengrui Pharmaceutical Co., Ltd., China in accordance with the guidelines of Good Manufacturing Practice (Chinese Edition). The manufacturer will have no direct involvement in the study (apart from the drug manufacturing and delivery to the clinical trial centers). An assessment will be conducted at the end of every second cycle of the described administration protocol, until unacceptable toxicity or disease progression is observed.

### Dose adjustment

Principle for dose adjustment: in the case of adverse events associated with apatinib, the dose will be adjusted first (Table 2). There are two dose levels for apatinib: (1) initial dose: 500 mg, once daily; (2) secondary dose: 250 mg, once daily. Medication will be paused if the patients cannot recover from drug toxicity. The time for each pause and the cumulative time of overall pauses per cycle are limited to 1 week. There is a maximum of two pauses per cycle, to ensure the medication intensity in each patient. Patients not meeting the above criteria, or having a delay of the subsequent treatment cycle of at least 2 weeks, will be required to withdraw from the trial.

**Table 2:** Principle for the adjustment of apatinib dose

Classification of adverse reactions	NCI-CTC level	Dose adjustment
Hematologic adverse reactions	3	Drug administration will be paused at level 3 and will be continued at the initial dose when the NCI level is restored to ≤ level 2. If NCI ≥ level 3 occurs again, drug administration at the secondary dose will be continued
	4	Drug administration will be paused at level 4 and will be continued at the secondary dose when the NCI is ≤ level 2
Non-hematologic adverse reactions	3	Drug administration will be paused at level 3 and will be continued at the initial dose when the NCI is restored to ≤ level 1. If the NCI ≥ level 3 occurs again, drug administration at the secondary dose will be continued
	4	Drug administration will be paused at level 4 and will be continued at the secondary dose when the NCI is ≤ level 1

Adverse reactions will be assessed using the National Cancer Institute Common Toxicity Criteria (NCI-CTC) Version 4^30^

The dose of apatinib can be adjusted at any time during each dosing cycle. Once the dose is reduced, however, a subsequent increase to the previous level is not permitted. Only a single dose adjustment is allowed for each subject. After the dose is decreased to 250 mg, no further dose adjustments are permitted for any reason. However, pausing of medication administration will still be permitted.

When any conditions that meet the criteria for drug withdrawal are observed, administration of vinorelbine will be discontinued. If the patient meets the criteria for drug re-administration in the subsequent cycles, vinorelbine administration will be resumed, but the doses that are not taken during the withdrawal period will be omitted (Table 3).

**Table 3:** Criteria for drug withdrawal and re-administration

**Drug withdrawal**	
Neutrophil	Level 3 or 4
Platelet	Level 3 or 4
Serum creatinine	1.5 times over the upper limit of normal value (44–132 μM)
Infection	Infectious fever with temperature > 38 °C
Diarrhea, mouth ulcers	Level 3 or 4
Others	When adverse reactions occur in addition to the above, and the investigators cannot judge whether drug administration can be continued or not, the drug will be discontinued
**Drug re-administration**	
Neutrophil	> 1.0 × 10^9^/L
Platelet	> 50 × 10^9^/L
Serum creatinine	< the upper limit of normal value (44–132 μM)
Infection	No infectious fever
Diarrhea, mouth ulcers	≤ Level 1
Others	When adverse reactions leading to drug withdrawal are alleviated or disappear, the administration can be resumed as determined by the investigators

Drug withdrawal and re-administration will be assessed using the National Cancer Institute Common Toxicity Criteria (NCI-CTC) Version 4^30^

### Concomitant medications

Conventional medications will be given to address patient symptoms. These may include prophylactic antiemetics and treatment with granulocyte colony-stimulating factor (as indicated by the patient’s hemogram). Hematopoietic growth factor support is permitted to avoid treatment interruption or delay. All symptomatic medications will be documented and detailed on a case report form.

### Assessment

#### Baseline evaluations (conducted within 2 weeks of the start of protocol therapy)

We will collect baseline data as follows:

- Demographic data
- American Joint Committee on Cancer tumor diagnosis and staging
- Relevant clinical disease history (diagnosis and treatment)
- Concomitant medication
- Physical examination
- Vital signs
- ECOG-PS score
- Imaging (computed tomography (CT) or magnetic resonance imaging (MRI))
- Electrocardiogram
- Laboratory tests: routine blood test, liver and kidney functional electrolytes (including K^+^, Na^+^, Cl^−^, Ca^2+^, Mg^2+^)
- Tumor markers: CA153, carcinoembryonic antigen (CEA)
- Pregnancy test (if necessary)
- The European Organization for Research and Treatment of Cancer Quality of Life Questionnaire Core-30 (EORTC QLQ-C30, version 3)

#### During treatment (conducted every 6 weeks (two cycles))

During treatment we will collect data on the following:

- Physical examination
- Vital signs
- ECOG-PS score
- Imaging test (CT or MRI)
- Laboratory tests: routine blood test, liver and kidney functional electrolytes (including K^+^, Na^+^, Cl^−^, Ca^2+^, Mg^2+^)
- Tumor markers: CA153, CEA
- EORTC-QLQ-C30 version 3 score
- Adverse reactions, concomitant medications

#### Follow up

At 4 weeks after treatment discontinuation we will collect data on the following:

- Physical examination
- Vital signs
- ECOG-PS score
- Laboratory tests: routine blood test, liver and kidney functional electrolytes (including K^+^, Na^+^, Cl^−^, Ca^2+^, Mg^2+^)
- Tumor markers: CA153, CEA
- EORTC-QLQ-C30 version 3 score
- Adverse reactions
- Concomitant medications (opioid analgesic consumption and new anticancer treatment)

Every 3 months after treatment discontinuation, until the patient dies or reaches study completion, we will collect data on the following:

- ECOG-PS score
- New anticancer treatment(s)
- Disease progression
- Survival status

### Outcomes

#### Primary outcome

PFS refers to the length of time from randomization and group allocation to any recorded tumor progression (including tumor recurrence, presence of new lesions, tumor treatment, and the use of other systemic or targeted anti-tumor therapies) or death (due to any cause). If the patient has several indicators that can be judged as progression of disease, the PFS analysis will be performed based on the indicator that first emerged. For patients with no tumor progression and who are still alive by the end of the study, the PFS time recorded at the last follow up will be censored. Tumor conditions during the treatment and follow-up periods will be evaluated in accordance with the RECIST version 1.1 criteria.

#### Secondary outcomes

The secondary outcomes are as follows:

1. OS refers to the length of time from randomization to mortality from any cause. In cases where no information on mortality is available in the clinical database, the last date on which the patient is known to have survived is used as the cut-off point.
2. The DCR indicates the percentage of patients who have achieved CR, partial remission (PR), and disease stabilization for over 4 weeks continuous, accounting for all the subjects with evaluable efficacy.
3. The ORR is the proportion of patients who achieve CR or PR ([CR + PR]/total number of cases × 100%), as assessed by the RECIST v1.1 [30].
4. Adverse events at levels 3 and 4: patients with adverse events at levels 3 and 4 will be assessed according to the National Cancer Institute Common Toxicity Criteria (NCI-CTC) Version 4.0 [32].

#### Other measures

Other measures will be performed as follows:

1. Quality of life will be assessed using the EORTC QLQ-C30 version 3.0 [33]. The scores will be averaged and transformed linearly to obtain a range of scores, from 0 to 100, with higher scores indicating better quality of life.
2. General health status is assessed using the ECOG-PS scale [34]. The scale divides the patient’s activity status into 0–5 levels. A higher level indicates a worse physical status.
3. Laboratory examination: (1) hematology assessments: hemoglobin, white blood cell count, neutrophil count, and platelet count; (2) blood biochemical tests: total bilirubin, ALT, AST, alkaline phosphatase, serum creatinine, total protein, Na^+^, K^+^, Mg^2+^, Cl^−^, Ca^2+^, urea, and pregnancy test (if applicable); and (3) tumor marker detection: CA153 and CEA.

### Adverse events

All adverse events will be recorded appropriately in a case report form and assessed by the investigators according to the NCI-CTC version 4.0 [32]. If the level of ALT or AST is ≥ 3 × ULN, or total bilirubin ≥ 2 × ULN, a serious adverse event (SAE) report may be necessary. The investigators must promptly determine whether the patient meets Hy’s Law (drug-induced liver injury (DILI)), without delay.

The investigators will assess the causal relationship between adverse events and target drugs. The decisive factor in the assessment is the temporal correlation between the adverse event and target drug. All deaths during the intervention period, and during the last follow-up period (30 days after the last dose) and the period up to documented disease progression (whichever occurs later) will be reported.

The investigator is obliged to immediately (within 24 h) call, fax, or email information on any serious or medically significant clinical adverse events or laboratory abnormalities during the study period, to the Adverse Drug Reaction Monitoring Center, the Sponsor, and the Ethics Committee. This applies regardless of group allocation. Optimal supportive therapy will be provided in cases of hematological toxicity, non-hemotoxic diarrhea, liver toxicity, and peripheral neurotoxicity.

### Criteria for re-administration/cycle delay

Patients who meet all of the following criteria can receive the planned treatment:

- Absolute neutrophil count (ANC) > 1500/mm^3^
- Platelets > 100,000/mm^3^
- Treatment related to non-hematologic toxicity has been eliminated at baseline or is at a level ≤ 1 (except for level-2 alopecia or level-2 fatigue)

If the patient cannot meet these criteria, the planned treatment will be delayed and the patient’s condition will be re-evaluated at least once a week.

Additional criteria for re-administration/cycle delay are as follows:

- The medication on the 8th day of each cycle cannot be delayed for longer than 1 week, otherwise, the patient will be withdrawn from the study. The time of medication administration in the next cycle will not change.
- In the case of failure to recover from treatment-related toxicity to baseline or level 1 (except level 2 and level-2 fatigue) within 3 weeks as scheduled (i.e., the start of each new cycle is delayed for over 21 days as compared with the scheduled time), the patient will be withdrawn from the trial.
- Patients can continue to use the drug, with the consent of the Sponsor, if the treatment is deemed to be effective.

### Participant withdrawal

Patients will be withdrawn from the study if they (1) withdraw their informed consent; (2) request to withdraw from the trial; or (3) decline to continue treatment or follow up. All source data and source files related to all withdrawn participants will be retained. The time and cause of withdrawal will be recorded on the case report form in detail.

### Monitoring

Progression of the trial, adverse events, and data quality will be monitored by an Independent Data (and safety) Monitoring Board (IDMB), which is independent of the trial Sponsor. The IDMB will be responsible for reporting the security data in the trial to the primary investigator. The primary investigator will submit a list of all suspected SAEs to the Independent Ethics Committee (IEC), as well as a summary of all reported SAEs every 6 months.

### Audits

An inspector will review the incoming data monthly and generate a data query if necessary. The inspector will review whether each electronic case report form is completed accurately. All discrepancies in the electronic case report form will be corrected by the investigator or authorized personnel in an appropriate manner.

### Data management

Data entry and management are the responsibility of an independent data administrator, who will use the EpiData 3.1 software (The EpiData Association, Denmark, Europe). All data will be independently inputted and proofread by two data administrators, to ensure accuracy. The data administrators will list the questions in the case report form in the data request queue (DRQ), and the investigator will respond as soon as possible. The data administrators will then modify, confirm, and enter the data according to the investigator’s responses. Another DRQ can be submitted if necessary. All original files will be kept in accordance with the principles of Chinese Good Clinical Practice, and clinical data will be kept by the investigators for 5 years (starting from the end of the clinical trial). All clinical data pertaining to the trial will be the property of the Sponsor, and the investigators will have no right to disclose these data to a third party without written approval by the Sponsor.

### Sample size

The survival time of the patients will be compared between the two groups by the log-rank test, using PASS Sample Size Software 11.0 (NCSS LLC, Kaysville, Utah, USA) [35]. Based on previous experience [36] and pilot study results, the mPFS was estimated to be 4.1 months in the vinorelbine monotherapy group and 6.7 months in the vinorelbine plus apatinib group (hazard ratio (HR) = 0.61). The recruitment time is expected to be 26 months (from the beginning of recruitment to the enrollment of the last patient) and a 15-month follow-up time is planned. Assuming a dropout rate of 10% across both groups, power of 80%, and two-sided significance of α = 0.05, a sample size of 238 patients (119 in each intervention group) will be required.

### Statistical analysis

The primary analysis will be based on the intent-to-treat (ITT) principle (i.e. all patients will be analyzed according to their allocated treatment group). Missing primary endpoint data will be imputed conservatively. We will consider a sensitivity analysis using complete case, i.e. not imputing missing values, to assess if there is a difference in results. No interim analysis will be performed.

A statistician will perform the statistical analyses using SPSS 22.0 software (IBM, Armonk, NY, USA). Continuous variables will be summarized as the mean, standard deviation, median, minimum, and maximum. Means and standard deviations will be reported if the variable is normally distributed; if the data are skewed, medians and interquartile ranges will be used. Categorical variables will be expressed as numbers and percentages.

The baseline data will be summarized accordingly, depending on the data type (continuous, categorical). Categorical variables will be compared between groups using Pearson’s chi-square test. Continuous variables will be compared between groups using the two-sample *t* test (normally distributed data) or Mann-Whitney U test (non-normally distributed data). All statistical analyses will be performed based on a two-sided test. A *P* value ≤0.05 will be considered statistically significant, and the 95% CI will be calculated.

Survival data (PFS and OS) will be estimated using the Kaplan-Meier method. Differences between grouped survival profiles will be assessed by the log-rank test. Factors influencing survival (i.e., age, tumor, node, metastasis (TNM) staging, tumor differentiation status, lymph node metastasis, and chemotherapy cycles) will be analyzed using Cox proportional hazard regression analysis. Results will be expressed as HRs and 95% CIs.

### Quality control

The clinical research unit will satisfy the requisite clinical research conditions for drug research, as determined by the National Medical Products Administration of China. Investigators will be clinically trained physicians who work under the direction of a senior physician. Clinical wards will be standardized to ensure that rescue equipment is fully functional. Medications will be administered by professional caregivers, and patient compliance will be ensured by learning about the medication. The standard operating rules of clinical trials will be followed and implemented in the research center. All clinical procedures will be supervised, and the complete and correct reporting of all data will be confirmed by comparing case report forms and the source data. In the event that an SAE occurs, it will be promptly reported to each research unit and, if necessary, the trial will be temporarily discontinued.

### Ethics and dissemination

#### Ethics and informed consent

The trial will be conducted in accordance with Good Clinical Practice guidelines, the guiding principles of the Declaration of Helsinki, and applicable local laws and regulations. The study protocol was approved by the IEC of the Liaoning Provincial Cancer Hospital in October 17, 2018 (approval No. 20180948–2) (Additional file 2) and the trial has been registered at ClinicalTrials.gov (identifier: NCT03932526). This research plan refers to protocol V2.0.

Written informed consent will be given by each patient prior to participation in the trial. The investigator will inform the patients that participation in the study is voluntary, and that they can withdraw at any time. Patients will be informed that the investigator will maintain their records over the long-term follow-up period, and that their records may be viewed by relevant management officers, within the limits of relevant laws and regulations. The privacy of all patients will be protected.

#### Dissemination

The final research results will be disseminated through publications in peer-reviewed academic journals and at international academic conferences.

### Protocol amendments

All amendments to the protocol will be signed and dated by the Department of Breast Medicine, the Liaoning Provincial Cancer Hospital, China, and approved by the IEC before release. Any protocol deviation that may occur during the study will be promptly managed by the investigator. The protocol deviation, including reason(s) for its occurrence, will be documented in the case report form and the original case report, which will be retained in the research unit by the Sponsor.

### Principle of confidentiality

Collection and use of patient data will comply with relevant laws and regulations that protect the subjects’ privacy, and full confidentiality will be maintained. The participants have the right to obtain their personal data through the investigator, and to modify any errors or incomplete data. No personal information will be disclosed to unauthorized parties. The Sponsor will maintain the confidentiality of all personal data throughout the study period.

### Compensation

The drugs used in this trial will be provided by the pharmaceutical company; the company has no role in the study design; collection, management, analysis, and interpretation of data; writing of the report; or the decision to submit the report for publication.

Each patient having provincial, municipal, remote, or new rural cooperation medical insurance will be subsidized with RMB 600 yuan (~ 85.5 USD) after completing one chemotherapy cycle. Self-paying patients will be given the subsequent cycle of chemotherapy free of charge (chemotherapy drugs only) after the initial cycle of chemotherapy.

Compensation mechanism: the specific compensation standards and methods will be clarified before the trial, and the Sponsor will provide clinical trial insurance for each subject. Treatment costs for drug-related adverse events and corresponding economic compensation will be undertaken by the Sponsor.

## Discussion

The anti-tumor effect of conventional chemotherapy relies on a single maximum tolerated dose. Despite its wide use, traditional scheduling of chemotherapy cycles is not convenient for patients, as frequent hospital visits for infusion and in-patient admission are required. To reduce the side effects of chemotherapy, treatment cycles are separated by longer intervals. This prospective randomized controlled trial, conducted in Liaoning Province of Northeast China, will be the first to analyze the efficacy of apatinib-targeted therapy, combined with vinorelbine chemotherapy, in treating patients with recurrent or metastatic TNBC, who have previously received anthracyclines and taxanes. Some limitations should be acknowledged in the current study protocol. As all subjects are limited to the northern Chinese population, the study results may not be generalizable to other patient populations. Furthermore, the sample size is relatively small, and this is due to limitations in staffing and funding.

Based on the data from this trial, we hope to identify an optimal treatment regimen that is suitable for female patients with TNBC in Northeast China, who have been treated with anthracyclines and taxanes.

### Trial status

The trial was registered at ClinicalTrials.gov (identifier: NCT03932526) on 30 April 2019. Patient recruitment will begin in June 2019, and end in August 2022. Analysis of the primary outcome measure will be completed in December 2022. The study will end in June 2023. This research plan refers to protocol V2.0.

## Supplementary information

**Additional file 1.** SPIRIT checklist.

**Additional file 2.** Ethics committee approval.

## Data Availability

The results of this study will be disseminated via peer-reviewed publications and conference presentations. No data are available at the moment.

## Abbreviations

AJCC: American Joint Committee on Cancer
ALT: Alanine aminotransferase
ANC: Absolute neutrophil count
AST: Aspartate aminotransferase
CEA: Carcinoembryonic antigen
CI: Confidence interval
CR: Complete response
CT: Computed tomography
DCR: Disease control rate
DILI: Drug-induced liver injury
DRQ: Data request queue
ECOG: Eastern Cooperative Oncology Group
ECOG-PS: Eastern Cooperative Oncology Group performance status
EDC: Electronic data capture system
EORTC QLQ-C30, version 3: European Organization for Research and Treatment of Cancer Quality of Life Questionnaire Core-30
ER: Estrogen receptors
HER-2: Human epidermal growth factor receptor 2
IDMB: Independent Data Monitoring Board
IEC: Independent Ethics Committee
mOS: Mean overall survival
mPFS: Mean progression-free survival
MRI: Magnetic resonance imaging
mTTP: Median time to progression
NCI-CTC: National Cancer Institute Common Toxicity Criteria
ORR: Objective response rate
OS: Overall survival
PR: Progesterone receptors
RECIST: Response Evaluation Criteria in Solid Tumors
SAEs: Serious adverse events
SPIRIT: Standard Protocol Items - Recommendations for Interventional Trials
TNBC: Triple-negative breast cancer
TTP: Time to progression
ULN: Upper limit of normal
VEGF: Vascular endothelial growth factor
VEGFR: Vascular endothelial growth factor receptor
