# Supplementary material for: Combined use of apatinib mesylate and vinorelbine versus vinorelbine alone in recurrent or metastatic triple-negative breast cancer: study protocol for a randomized controlled clinical trial
Source: Trials. 2020 May 24;21:420. doi: 10.1186/s13063-020-04342-x (PMC7245760; doi:10.1186/s13063-020-04342-x)
Supplement: Supplementary file 2 — Additional file 2. Ethics committee approval. [file 13063_2020_4342_MOESM2_ESM.pdf]

Application form for ethic approval

Approval No. 20180948-2

<table>
<tr><td>Research project & protocol version</td><td colspan="3">Combined use of apatinib mesylate and vinorelbine versus single use of vinorelbine in recurrent or metastatic triple-negative breast cancer: study protocol for a randomized controlled clinical trial

(Research project with a retrial after revision)</td></tr>
<tr><td>Sponsor</td><td>Liaoning Cancer Hospital & Institute</td><td>Setting</td><td>Liaoning Cancer Hospital & Institute</td></tr>
<tr><td>Researcher(s)</td><td>Tao Sun</td><td>Division</td><td>Department of Medical Oncology</td></tr>
<tr><td>Date of censors</td><td>2018.10.16</td><td>Setting</td><td>The meeting room of the Ethics Committee of Liaoning Cancer Hospital & Institute</td></tr>
<tr><td>Ethical review manner</td><td colspan="3">☐ Meeting review ✓
☐ Fast review</td></tr>
<tr><td>Documentation</td><td colspan="3">See attachment</td></tr>
<tr><td>Voting</td><td colspan="3">Anticipated: 15 Actually voted: 14
Abstained: 0 Avoided: 0</td></tr>
<tr><td>Review comments</td><td colspan="3">On October 16, 2018, the Ethics Committee conducted a serious discussion on the above-mentioned protocol and voted on it. There were 14 voters: 14 voting for the protocol, 0 agreeing on the protocol after necessary amendments, 0 agreeing on re-check after necessary amendments, and 0 voting against the protocol. The Ethics Committee believes that the protocol is basically in line with ethical requirements and can be conducted as a clinical trial.</td></tr>
<tr><td>Other suggestions</td><td colspan="3">Nil.</td></tr>
<tr><td>Period of validity</td><td>2018.10.17 - 2019.10.16</td><td>Periodic review</td><td>☐ 3 months
☐ 6 months
☐ 12 months ✓</td></tr>
<tr><td>Decision</td><td colspan="3">Agreement

Signature:____________
October 17, 2018</td></tr>
</table>

Note: The responsibilities, personnel composition, functions, operating procedures and records of the Ethics Committee follow ICH-GCP and relevant laws and regulations in China.

Researcher: _________________ Date:________________

Sponsor: ____________________ Date: _______________

辽宁省肿瘤医院医学伦理委员会审查批件

伦理批件编号：20180948-2

项目名称及版本号	甲磺酸阿帕替尼联合长春瑞滨软胶囊对比长春瑞滨软胶囊用于一线及以上三阴性乳腺癌的多中心、随机对照 II 期临床研究 （科研项目，修正后重审）		
申办/参研单位名称	辽宁省肿瘤医院	临床试验机构	辽宁省肿瘤医院
研究者	孙 涛	研究所在科室	乳腺内科病区
审查日期	2018.10.16	审查地点	辽宁省肿瘤医院伦理委员会会议室
伦理审查方式	✓☐会议审查 ☐快速审查		
审查文件	详见附件		
投票情况	应到人数：15 人 实到人数：14 人<br>弃权人数：0 人 回避委员：0 人		
审查意见	伦理委员会于 2018 年 10 月 16 日对上述方案及有关内容进行了认真的讨论并进行了投票表决。投票人数：14 人，同意 14 票，作必要的修正后同意 0 票，作必要的修正后重审 0 票，不同意 0 票。伦理委员会认为该项目资料基本符合伦理要求，可以开展临床研究。		
其他意见或建议	无		
批件有效期	2018.10.17-2019.10.16	跟踪审查频率	☐3 个月 ☐6 个月 ✓☐12 个月
决 议	同 意	伦理委员会主任/授权者签字：[signature]<br>2018 年 10 月 17 日	

注：本伦理委员会的职责、人员组成、作用、操作程序及记录遵循 ICH-GCP 和中国的相关法律法规。

1.临床研究人员签收：孙涛　　　　日期：2018.10.30

2.申办单位人员签收：________　　　　日期：________

地址:辽宁省沈阳市大东区小河沿路 44 号 邮编:110042 电话/传真:024-31916632 e-mail: szlyy315@163.com
